# Study of Cannabis Oils Obtained from Three Varieties of *C. sativa* and by Two Different Extraction Methods: Phytochemical Characterization and Biological Activities

**DOI:** 10.3390/plants12091772

**Published:** 2023-04-26

**Authors:** Sebastián Pino, Luis Espinoza, Carlos Jara-Gutiérrez, Joan Villena, Andrés F. Olea, Katy Díaz

**Affiliations:** 1 LABSUN (Laboratorio Sustentable Natural), Valparaíso 2340000, Chile; sebastian.pino@alumnos.usm.cl; 2 Departamento de Química, Universidad Técnica Federico Santa María, Avenida España 1680, Valparaíso 2340000, Chile; luis.espinozac@usm.cl; 3 Laboratorio de Investigación-Estrés Oxidativo, Centro de Investigaciones Biomédicas (CIB), Facultad de Medicina, Universidad de Valparaíso, Viña del Mar 2520000, Chile; carlos.jara@uv.cl (C.J.-G.); juan.villena@uv.cl (J.V.); 4 Grupo QBAB, Instituto de Ciencias Químicas Aplicadas, Facultad de Ingeniería, Universidad Autónoma de Chile, El Llano Subercaseaux 2801, Santiago 8900000, Chile

**Keywords:** marijuana, cannabinoids, cytotoxic effect, medical oils

## Abstract

Currently, much effort is being placed into obtaining extracts and/or essential oils from *Cannabis sativa* L. for specific therapeutic purposes or pharmacological compositions. These potential applications depend mainly on the phytochemical composition of the oils, which in turn are determined by the type of *C. sativ*a and the extraction method used to obtain the oils. In this work, we have evaluated the contents of secondary metabolites, delta-9-tetrahydrocannabinol (THC), and cannabidiol (CBD), in addition to the total phenolic, flavonoids, and anthraquinone content in oils obtained using solid–liquid extraction (SLE) and supercritical fluid extraction (SCF). Different varieties of *C. sativa* were chosen by using the ratio of THC to CBD concentrations. Additionally, antioxidant, antifungal and anticancer activities on different cancer cell lines were evaluated in vitro. The results indicate that oils extracted by SLE, with high contents of CBD, flavonoids, and phenolic compounds, exhibit a high antioxidant capacity and induce a high decrease in the cell viability of the tested breast cancer cell line (MCF-7). The observed biological activities are attributed to the entourage effect, in which CBD, phenols and flavonoids play a key role. Therefore, it is concluded that the right selection of *C. sativa* variety and the solvent for SLE extraction method could be used to obtain the optimal oil composition to develop a natural anticancer agent.

## 1. Introduction

*Cannabis sativa* L. (Cannabaceae), has been widely cultivated due to its industrial [1,2], ornamental [3], nutritional [4,5], medicinal [6,7], and recreational [7] potentials. From regulatory and application perspectives, *cannabis* plants are categorized based on the level of Δ-9-tetrahydrocannabinol (Δ9-THC or simply THC), one of the most important phytocannabinoids [8,9,10]. Plants are generally classified and regulated as industrial hemp considering the THC content in the dried flower. Legislation in the EU has established a 0.2% THC threshold ((EC) No 2860/2000) to differentiate fiber hemp from drug-type *Cannabis* [11]. Thus, in recent years, an increasing number of countries are regulating its recreational and medical use [12,13,14].

The phytochemistry of *C. sativa* L. has been extensively studied, and nearly 500 compounds have been identified [9,15]. Most of them are secondary metabolites, and the most specific class of cannabis constituents are the C21 terpenophenolic cannabinoids. From these, THC, responsible for the psychotropic effects [16], and cannabidiol (CBD) are the predominant forms of cannabinoids in drug-type and fiber-type varieties of cannabis, respectively. Other phenolic components of cannabis include flavonoids, spiroindanes, dihydrostilbenes, phenanthrenes and dihydrophenanthrenes [15,17,18,19,20,21]. The concentration of these compounds depends on many factors such as plant variety, growth conditions and harvest time. Since these compounds are secondary metabolites, external factors such as climatic conditions, geographical origin, harvesting time and extraction procedure can significantly affect their content and extract quality in *C. sativa* L. [22], and the distribution of these molecules within the plant can vary the final composition [23]. However, *C. sativa* is a single species, and hundreds of cultivars have been developed for increasing or decreasing THC content [24]. There have been some attempts to classify cultivars based on chemical composition [19,25,26,27], and different effects of cannabis have been attributed to different values of the ratio of THC/CBD [28].

Both extracts and compounds isolated from different parts of the cannabis plant have shown antifungal, antibacterial, antioxidant, antimalarial, antileishmanial, cytotoxic and pharmacological effects [18,29,30,31]. Numerous studies have determined the effect of Δ9-THC and CBD as anticonvulsants, analgesics, anxiolytics and antiemetics [32]. Both cannabinoids are metabolized in the liver by cytochrome P450 [32,33]. In the last decade, advocates of medicinal cannabis have demonstrated its potential for the treatment of various diseases, including cancer [34,35]. Cannabinoids are known to have palliative effects on cancer patients [10], helping to reduce the sensations of nausea, pain and vomiting induced by chemotherapy, as well as helping with insomnia and appetite [36]. Interestingly, cannabinoids have proven to selectively inhibit the in vitro growth of cancer cell lines and in vivo tumors in animal models [37].

In recent decades, research on the biological activity of *C. sativa* has been focused on extracts or oils of the whole plant (full spectrum), in which mixtures of cannabinoids and natural terpenes are present [38]. This approach has been used to obtain complex mixtures of compounds or essential oils from aromatic plants [39,40,41], and it has been demonstrated that their biological activities depend on the relative concentration of their components [42]. In a similar way, it has been found that *cannabis* oils exhibit higher activity than isolated compounds, and this synergic effect is known as the entourage effect [34,43]. Therefore, the development of pharmacological and biotechnological applications based on antioxidant and antimycobacterial properties will strongly depend on the extraction procedure [2], which can significantly modify the relative composition of oils obtained from *C. sativa* L. [22].

Currently, solid–liquid extraction (SLE) with organic solvents is the most widely used method for medical applications of cannabis [4,44,45,46]. However, more recent studies have shown that supercritical fluid extraction (SCF), which avoids the use of potentially toxic organic solvents, produces higher quality extracts and yields at shorter operating times [4,46,47,48].

Therefore, in this study, these two extraction methods have been used to obtain cannabis oils from different plant varieties of *C. sativa* L. inflorescence. The obtained oils were submitted to a phytochemical study to quantify the presence of secondary metabolites, including Δ9-THC and CBD, and the total content of phenols, flavonoids and anthraquinones. Then, various important biological activities of these oils were evaluated, such as antioxidant capacity, antifungal activity against *T. mentagrophytes* and in vitro cytotoxicity on different cancer cell lines, i.e., HT-29 colon cancer, MCF-7 breast cancer and PC-3 human prostate cancer. Potential correlations between phytochemical data and biological activities are discussed.

## 2. Results and Discussion

In this work, three plant varieties of *C. sativa* L. were used, which, according to the supplier, gives different ratios of Δ9-THC/CBD in the plant inflorescences. These are Critical+, Shark Shock CBD, and Dinamed CBD with THC/CBD ratios equal to 1:0, 1:1 and 0:1, respectively. Seeds were cultivated under controlled conditions (see Material and Methods) and after harvest of cannabis inflorescence the plant material was extracted by SLE using ethanol [49], or SCF using supercritical CO_2_ (SC-CO_2_). The latter is a green technique, and it is widely used to obtain bioactive compounds at large scale [23,45,50,51]. In this way, six cannabis essential oils were obtained with similar yields for all varieties and both extraction methods (10–13 wt%) (see Table 1).

### 2.1. Phytochemical Study

#### 2.1.1. Chromatographic Analysis of Essential Oils by HPLC-UV

Separation and quantification of main cannabinoids, Δ9-THC and CBD, in plant inflorescence extracts was carried out by High Performance Liquid Chromatography coupled to ultraviolet spectroscopy (HPLC-UV) and using a Chromolith RP-18 column [52,53,54,55] (Table 1; Supplementary material Figure S1).

Results shown in Table 1 indicate that for varieties Critical+ and Shark Shock CBD (ratios 1:0 and 1:1, respectively) extraction with SC-CO_2_ gives slightly higher or similar amounts of THC, whereas the CBD content is higher for the SLE method. On the other hand, for extracts obtained from variety Dinamed CBD (ratio 0:1) both THC and CBD content were much higher for SC-CO_2_ extraction. This result is in line with previous work where it has been shown that SC-CO_2_ extraction leads to higher CBD yields [44,56,57]. However, the measured THC/CBD ratios are very similar to those given by the supplier, excepting Shark Shock CBD whose ratio should be 1:1 and for both extraction methods the relative amount of CBD is higher than that of THC (ratios 0.72:1.0 and 0.58:1.0). This is an interesting result because diverse pharmacological activities of CBD have been described [57] and therefore these extracts are good candidates for exploring potential medical applications. Considering that all *C. sativa* varieties were grown under the same cultivar conditions it becomes clear that differences in detected amounts of cannabinoids are due to the used extraction procedures. This conclusion is in line with previous report where it has been proposed that differences in cannabinoids content can be attributed to preferential extraction instead of agrotechnical conditions [52]. For varieties with similar content of these compounds (ratio 1:1) any one of these tested methods will give similar results.

It is worth to emphasize that the extraction method of choice depends on the desired properties of the cannabis oil.

#### 2.1.2. Evaluation of Total Content of Phenols, Flavonoids and Anthraquinones

It has been proposed that the entourage effect is mainly due to the interaction between cannabinoids and a myriad of different compounds existing in cannabis oils, such as terpenes, flavonoids and phenolic compounds [28,43]. It is well established that in many cases the pharmacological activity of pure THC or CBD is lower than that exhibited by cannabis extracts because many of the minor components have their own pharmacological activity [43]. For example, it has been shown that phenolic compounds are responsible for the antioxidant properties of *C. sativa* extracts [58] and that some flavonoids present anticancer activities [59]. Therefore, to compare the biological activities of the extracts obtained from different varieties of *C. sativa* L., the total content of phenols, flavonoids and anthraquinones were determined by colorimetric methods (see Material and Methods). Briefly, the concentration of phenols, flavonoids and anthraquinones were obtained by spectrophotometric measurements using the absorbance calibration curves of gallic acid, quercetin and emodin, respectively. The results in Table 2 are given as the equivalents of these standards (mg/g of dried oil).

The data in Table 2 indicate that the total content of phenolic compounds is independent of the extraction method, but it is slightly higher in oils obtained from Critical+ (M1 and M2) and Shark Shop CBD (M3 and M4) than in oils from Dinamed CBD (M5 and M6). In other words, the highest amounts of phenolic compounds are found in *C. sativa* varieties with the highest content in Δ9-THC (ratios 1:0 and 1:1). Thus, it could be suggested that the formation of phenolic compounds and Δ9-THC are somehow related. On the other hand, the total content of flavonoids varies with the extraction method; i.e., the SLE method gives concentration values in the order of 6.32–9.10 QE mg/g (M2, M4 and M6) that are almost twofold the values found for SC-CO_2_ (M1, M3 and M5). It is known that in the SLE method the extract composition will depend on the polarity of the solvent used. In this case, ethanol is more polar than CO_2_, and, therefore, the extraction of polar flavonoids is enhanced in the SLE method. Finally, the content of anthraquinones (in the order of 5.00 to 6.19 EE mg/g) is independent of the extraction method and the variety of *C. sativa* L.

Therefore, the samples extracted by the SLE method (M2, M4 and M6) contain the highest concentration of phenolic compounds and flavonoids. Additionally, M4 (THC:CBD ratio 0.58) is one of the samples with the highest content of THC and CBD.

### 2.2. Assessment of Biological Activities

#### 2.2.1. Evaluation of Antioxidant Capacity

Antioxidant capacity is a term used in biological systems to describe the protective action of compounds against oxidative degradation induced by reactive oxygen species (ROS). The mechanism of action includes a variety of different processes, and, therefore, no single assay can measure the antioxidant capacity of all antioxidants in a complex system. The various methods that have been developed can be classified according to two main mechanisms: hydrogen atom transfer (HAT) and single electron transfer (SET). In HAT methods, the radical is deactivated by the hydrogen atom donation from the antioxidant compound, whereas, in ET methods, the radical is deactivated by the transfer of one electron from the antioxidant. Thus, the antioxidant capacity of all extracted oils was assessed by using three common assays: 1,1-diphenyl-2-picryl-hydrazyl (DPPH), 2,2′-azino-bis-3-ethylbenzothiazolin-6-sulphonic acid (ABTS), and ferric-reducing antioxidant power (FRAP). The results obtained for all of the cannabis oil samples are listed in Table 3 and are expressed in different units depending on the used method: IC_50_ (mg/mL) for DPPH; mM of Trolox equivalent antioxidant capacity (TEAC) for ABTS and FRAP.

The results show that, in the ABTS test, the M4 extract registers a significant average value close to the unity. In this test, the radical cation, *ABTS^+^* reacts with phenolic compounds by the H-atom transfer, and the consumed amount is expressed in Trolox equivalents (TEAC). It has been shown that TEAC for Trolox is equal to 1 [60], and, therefore, the TEAC values obtained for cannabis oils M2-M6 suggest that their antioxidant capacity is mainly due to the HAT mechanism. These H-transfer processes generally involve flavonoids and phenolic compounds.

This conclusion is supported by the results obtained with the FRAP assay, which indicate that all of the different cannabis oils show no antioxidant activity by electron transfer compared to the positive control substances. The FRAP assay cannot detect compounds that scavenge radicals by the H-transfer.

Finally, the antioxidant capacity determined by the DPPH test is measured by the concentration needed to decrease the initial DPPH concentration by 50%. The data are calculated as mean inhibitory concentration (IC_50_), and this means that the lowest values of IC_50_ correspond to the highest antioxidant capacity. Thus, the values in Table 3 indicate that the most active extracts are M1 and M4.

It is worth emphasizing that these three methods determine the antioxidant capacity via indirect reactions; i.e., they measure the ability to react with the ABTS radical cation, the DPPH radical, or reducing Fe (III). Consequently, there exists many interferences that can affect the results, and this has been discussed extensively elsewhere [60,61]. However, in this work, these methods have been used to compare the antioxidant capacity of a series of cannabis oils under the same experimental conditions. Thus, the differences found in the antioxidant capacity can be attributed exclusively to the phytochemical composition of these oils. It is interesting to note that samples with different concentrations of the main cannabinoid’s exhibit similar antioxidant activity. Therefore, it can be concluded that this property is a consequence of the action of many different compounds, i.e., flavonoids and phenolic compounds. Antioxidant capacity can, thus, be explained as the result of a synergic effect between THC, CBD and phenolic types of metabolites that are present in these cannabis oils [62]. These results are in line with previous works in which important differences on these effects have been reported for *Cannabis* with various ratios of THC/CBD [28].

#### 2.2.2. Evaluation of Antifungal Activity

The antifungal activity of M1-M6 was tested against *Trichophyton mentagrophytes*, which is an anthropophilic, pathogenic fungus that causes tinea capitis of the feet and body and invades the nail surface of both humans and animals [63,64]. The results show that the EC_50_ values for cannabis oils are in the range of 89–240 μg/mL (see the last column in Table 3). The lowest values of EC_50_ were obtained for samples extracted by the SEL method, and M4 stands out with the highest antifungal activity (EC_50_ = 89.4 μg/mL). This could be attributed to M4 having the largest concentrations of phenolic compounds and flavonoids obtained by extraction with ethanol (see Table 2). Interestingly, a comparison of antifungal activities between samples 4 and 5 shows that a tenfold decrease in THC, but small changes in total phenolic and flavonoid content (Sample 5) induce a small change in EC_50_. These results suggest that the antifungal activity of cannabis oils is due to the action of several different compounds. This is in line with a study in which twelve dermatophytes were treated with ethanol extracts of two different cannabis strains [65]. Finally, the EC_50_ values of M4-M6 are lower than that shown by fluconazole, a commercial antifungal agent. Thus, essential oils emerge as therapeutic alternatives for dermatophytosis and likely other types of fungal infections.

#### 2.2.3. Evaluation of In Vitro Cytotoxicity against Different Cancer Cell Lines

The cytotoxicity of cannabis oils (M1–M6) was evaluated in vitro against different cancer cell lines: HT-29 colon cancer, MCF-7 breast cancer and PC-3 human prostate cancer. To assess their toxicity against normal cells, a non-tumoral cell, MCF-10A, was used as the control. The colorimetric sulforhodamine B (SRB) assay was used to estimate the cell viability of cancer cell lines in the presence of different concentrations of cannabis oil (from 15 to 300 µg/mL).

From a plot of percentage cell viability as a function of cannabis essential oil concentrations, the IC_50_ values were obtained by fitting the data to a dose–response equation. The curves obtained for the most active samples (M2, M4 and M6) are shown in Figure S2 in the Supplementary Material, and all IC_50_ values are listed in Table 4.

On the other hand, the selectivity index (SI), which gives the selectivity of extracts acting on cancer cells with respect to their effect on a normal cell line, can be calculated as the ratio between the IC_50_ value obtained for the control cell line, MCF-10A, and the IC_50_ values calculated for tumor cell lines (MCF-7, HT-20 and PC-3). If this number is greater than 2, it means that the oil cytotoxicity is highly selective; i.e., the oil effect on tumor cells is larger than on normal cells. These SI values are also listed in Table 3.

The data indicate that the IC_50_ values of cannabis oils range from 13.0–64.2 μg/mL for cancer cell lines and that M2, M4 and M6 exhibit the highest cytotoxicity (the lowest IC_50_ value). At the same time, the corresponding SI index of the most active cannabis oils is between 2 and 5. For example, the M4 cannabis oil presented the lowest IC_50_ value (13 µg/mL) and the highest SI value on the cytotoxicity assay on the breast cancer cell line, MCF-7. In other words, M4 is the most cytotoxic and selective extract acting on this cell line. In addition, the M2, M4 and M6 samples also present important cytotoxic and selective activity on all cancer cell lines. The selective action of cannabinoids on tumor cells have been reported, but the mechanism remains unclear [37]. Thus, it can be concluded that the cannabis oils obtained by the LSE extraction method (M2, M4 and M6) exhibit higher cytotoxicity on breast, colon and prostate cancer cell lines than those obtained by the SCF method. These striking differences on cytotoxicity can be explained in terms of the chemical composition of oils obtained by LSE or SCF extraction. The data in Table 1, Table 2 and Table 3 indicate that oils obtained from the same plant variety, but using different extraction methods, give completely different phytochemical composition. Thus, the amounts of THC detected are similar in both extracts (Table 1); however, at the same time, the CBD and flavonoid concentrations are higher in the SLE extracts (Table 1 and Table 2). These results suggest that the cytotoxic effect of cannabis oils is mainly due to the presence of CBD, flavonoids, and likely other phenolic compounds, which act together via the entourage effect. Previous studies have shown that CBD induces cell death in breast [66,67,68,69] and lung [70] cancer cell lines, whereas its parent molecule, cannabidiolic acid CBD-A, inhibits breast cancer cell migration via a mechanism that inhibits protein kinase [71]. It has also been demonstrated that some common flavonoids, such as quercetin and luteolin, reduce the cell proliferation of MCF-7 [59,72], and other specific flavonoids of *C. sativa*, cannflavins, show anticancer activities as well [62]. Prenylated cannflavins have shown anticancer activity in vitro on human breast cancer cell lines [73] and in vivo against pancreatic tumors in animals [74].

Therefore, it can be concluded that the composition of cannabis oils extracted using the SLE method can be modulated to obtain a CBD and flavonoids mixture with the optimal entourage effect to create an antiproliferative effect on cancer cell lines.

## 3. Materials and Methods

### 3.1. Plant Material and Growing Conditions

The plant varieties of *C. sativa* L. selected for this experiment were selected from the seed bank Dinafem Seeds (Gipuzkoa, Spain), with specimens grown from cuttings to maintain genetic stability: Critical+ with 1:0 ratio (inflorescences with high Δ9-THCA content), Shark Shock CBD with 1:1 ratio (inflorescences with balanced content of both CBD-A and Δ9-THCA), and Dinamed CBD with 0:1 ratio (inflorescences with high CBD-A content).

All varieties of *C. sativa* L. were cultivated indoors using the research facilities of LABSUN company (Valparaíso, Chile) and under the same environmental conditions. Briefly, in two 80 cm^2^ plots, indoors, four plants were each placed in 11 L pots with BIOBIZZ^®^ ALLMIX substrate, together with a 300 w LED panel light system and a traditional 250 w high pressure lamp. BIOBIZZ nutrient line was used for growth during the first month (BIOGROW, ALGA-MIC). Then, BIO-BLOOM, BIO-HEAVEN and TOP-MAX were used for flowering for three months. Irrigation water was tested for electroconductivity (ranges from 600 to 2000 ms (0.6~2.0 EC) and pH (5.8–6.8)). From each growing season, 100 g of dried inflorescences were obtained during August.

### 3.2. Extraction

Six cannabis oils were obtained using two extraction methods, namely, solid–liquid extraction using ethanol 96% [49] and extraction by SCFE using supercritical CO_2_ (SC-CO_2_). In the former method, 100 g of inflorescences were preheated in an oven at 120 °C for 30 min to decarboxylate the acidic cannabinoids THCA and CBDA [49,75]. Then, the plant material was crushed, and 500 mL of ethanol were added. The mixture was shaken by 3 min and filtered, and the solvent evaporated under reduced pressure [76]. Supercritical fluid extraction was performed using a SuperC extractor (OCO Labs, Sierra Vista, AZ, USA). Briefly, the CO_2_ flow rate was 1.5 lb/h and the extraction process was carried out at 250 bar at 63 °C for1 h.

### 3.3. Phytochemical Study

#### 3.3.1. HPLC Analysis

A Young YL 9110 Plus HPLC coupled with diode array detector was used to separate and quantify the cannabinoids THC and DPA. The separation was performed with a two column in series array (Chromolith RP-18e, Merck). Mixtures of water (A)/acetonitrile (B) were used as mobile phase at flow rate of 2 mL/min. The separation was obtained with the following gradient: 0–3 min 20% B; 3–10 min 60% B; 10–11 min 95% B. The injection volume was 20 µL, and the detection was set at 211 nm [53,55].

#### 3.3.2. Total Phenolic Content Determination

The total phenolic content in the extracts was determined by using the Fiolin–Ciocalteau assay [77,78,79]. Cannabis extracts (2.0 mg) were dissolved in ethanol (2.0 mL), and 500 µL of each solution was mixed with Folin–Ciocalteau reagent (2.5 mL, 0.2 N) and incubated for 5 min. Then, a Na_2_CO_3_ solution (2.0 mL, 7.5% *w*/*v*) was added and incubated in the dark at room temperature for 2 h. The absorbance of the solution at 700 nm was measured against ethanol in a spectrophotometer (RayLEIGH, UV-2601, Beijing China). Absorbance values were converted to concentration using a gallic acid calibration curve (0–200 mg/L), and, therefore, total phenolic content was expressed as mg of gallic acid equivalents (mg GAE) per g of dried extract. All measurements were replicated three times.

#### 3.3.3. Total Flavonoid Content Estimation

A modified version of the Dowd method was used to determine the total flavonoid content [80]. Aluminum trichloride (AlCl_3_) in ethanol (1 mL, 2% *w*/*v*) was mixed with a dissolution of each extract in ethanol (1.0 mg/mL). The mixture was incubated for 10 min at room temperature, and absorbance was measured at 415 nm against a blank sample consisting of 1.0 mL of extract solution with 1.0 mL of methanol without AlCl_3_. Absorbance values were converted to concentration using a quercetin calibration curve (0–100 mg/L). The total flavonoid content was expressed as mg of quercetin equivalents (mg QE) per g of dry extract. All the measurements were replicated three times.

#### 3.3.4. Total Anthraquinones Content Estimation

Anthraquinones content was determined by using the following protocol [79,80]. Aluminum trichloride (AlCl_3_) in ethanol (1 mL, 2% *w*/*v*) was mixed with a dissolution of each extract in ethanol (1.0 mg/mL). The mixture was incubated for 10 min at room temperature, and absorbance was measured at 486 nm against a blank sample consisting of 1.0 mL of extract solution with 1.0 mL of methanol without AlCl_3_. Absorbance values were converted to concentration using an emodin calibration curve (0–70 mg/L). The total anthraquinones content was expressed as mg of emodin equivalents (mg EE) per g of dry extract. All measurements were performed in triplicate.

### 3.4. Evaluation of Biological Activities

#### 3.4.1. Measurement of Antioxidant Capacity

##### ABTS Assay

Several modifications of the original method [81] have been proposed; in this work, we have used the method developed by Romay et al. [82]. Briefly, one volume of a 10 mM solution of ABAP (2,2′-azo-bis (2-amidino propane) was mixed with the same volume of a 150 μM solution of ABTS (2,2′-azinobi (3-ethylbenzothiazoline-6-sulphonic acid) using PBS 100 mM at a pH of 7.4 (TRAP solution). The mixture was incubated at 45 °C for 30 min and then cooled down to room temperature. Sample solution (10 μL, 1.0 mg/mL of each extract) was mixed with the TRAP solution (990 μL), and the absorbance of the ABTS radical cation was measured after 50 s at 734 nm against the ABTS solution used as a reference. The absorbance values were interpolated in a Trolox standard curve (0–200 mg/L), and the results were expressed in mM Trolox equivalent antioxidant capacity (mM TEAC). All measurements were replicated three times.

##### DPPH Assay

Free radical scavenging capacity of oils was determined following the method proposed by Brand-Williams and modified by Miliauskas [83,84]. Samples of oils (M1–M6) were dissolved in ethanol (0–10 mg/mL), and 100 μL of each solution was mixed with DPPH solution (2.9 mL, 50 μM) freshly prepared in ethanol. A mixture prepared with 100 μL ethanol was used as control. Samples and control solutions were incubated for 15 min at room temperature, and the absorbance of DPPH radicals was measured at 517 nm. Radical scavenging activity was calculated by the following equation:

%DPPH radical scavenging = [(A_C_ − A_S_)/A_C_] × 100 (1)

where A_C_ and A_S_ are the absorbances of the control and oils, respectively. The plotting of the obtained data against cannabis oil concentration and the fitting of this data to the dose–response equation provide the IC_50_ values that are listed in Table 4.

##### Ferric Reducing Antioxidant Power (FRAP) Assay

The FRAP assay was performed using the following protocol [38]. Freshly prepared TPTZ reactive (10 volumes of 300 mM acetate buffer, pH 3.6) with 1.0 volume of 10 mM TPTZ (2,4,6-tri(2-pyridyl)-s-triazine) in 40 mM hydrochloric acid, and 1.0 volume of 20 mM ferric chloride FRAP reagent (3.0 mL) was mixed with deionized water (300 μL) and the sample (100 μL, 1.0 mg/mL of each extract). The mix was incubated for 30 min at 37 °C in a water bath, and the absorbance was measured at 593 nm using ethanol as reference solution. The obtained absorbance values were interpolated in a Trolox calibration curve (0–200 mg/L), and results were expressed in mM Trolox equivalent antioxidant capacity (mM TEAC). All measurements were performed in triplicate.

#### 3.4.2. Antifungal Assays

The tests were performed with *Trichophyton mentagrophytes* (CCCT 18.262), fungi, which is able to produce dermatophytosis or tinea infections. The isolate was obtained from “Collection Chilena de Cultivos Tipo” of the Universidad de la Frontera (CCCT-UFRO/BIOREN) and grown on dextrose potato agar (PDA) for two weeks at 28 °C to obtain the inoculum. The pure culture was stored in the pathogen collection of the Biological Testing Laboratory of the Chemistry Department of Universidad Técnica Federico Santa María.

Antifungal activity of all essential oils was evaluated by determining mycelial growth inhibition of *T. mentagrophytes* in the radial growth test [85].

All tested cannabis oils were dissolved in ethanol and water and added to potato dextrose agar (PDA) at 50 °C, reaching final concentrations ranging from 1 to 240 µg/mL. After solidification, Petri dishes were inoculated with 4 mm diameter agar discs containing fine mycelium of *T. mentagrophytes.* The negative control contained only PDA culture medium, and FLUCONAZOLE^®^ (IPhSA, INTHERFARMA S.A) was used as positive control. Three replicates were made for each treatment. All plates were incubated at 28 °C in the dark. After 7 days, the diameter of mycelial growth was measured and the percentage of mycelial inhibition (%I) was calculated. These values were plotted against fungicide concentration and fitted to a dose–response equation. This fit gives the concentration at which mycelial growth is inhibited by 50% compared to the negative control (EC_50_). Data plotting, fitting, and the calculation of EC_50_ values were carried out with Origin 8.0. Significant differences were assessed with a two-way analysis of variance (Tukey’s test; *p* < 0.05).

#### 3.4.3. Cancer Cells Cytotoxicity

##### Cultured Cell Lines

The following experimental cell lines were obtained from the American Type Culture Collection (Rockville, MD, USA): MCF-7 (human breast cancer; ATCC N°. HTB-22), HT-29 (human colon cancer; ATCC N°. HTB-38), PC-3 (human prostate cancer ATCC N°. CRL-1435) and MCF-10A (breast epithelial cell ATCC N° CRL-10317). All cell lines were grown in a DMEM-F12 medium containing 10% FCS, 100 U/mL penicillin, 100 μg/mL streptomycin and 1 mM glutamine.

##### In Vitro Cytotoxicity Screening Using Sulforhodamine B Assay

Sulforhodamine B assay was performed according to previously reported methods [86,87]. Cells were seeded into a 96-well flat-bottomed 100 μL microplate with a plating density of 3 × 10^3^ cells/well. After a 24 h incubation at 37 °C (under a 5% humidified carbon dioxide atmosphere to allow cell attachment), stock solutions of cannabis oil were prepared in ethanol and added to the growth medium to reach final concentrations (5–100 μg/mL) Negative controls were prepared by adding ethanol, and the final concentration of this solvent was kept constant at 1%. Cells were treated with different concentrations of extracts and incubated for 72 h under the same conditions. All culture microplates were incubated at 37 °C in a CO_2_ incubator with 5% humidified CO_2_ for 72 h. At the end of the extract’s exposure, cells were fixed with 50% trichloroacetic acid at 4 °C. After washing with distilled water, cells were stained with 0.1% SRB, dissolved in 1% acetic acid (50 μL/well) for 30 min, and then washed with 1% acetic acid to remove unbound stain. Protein-bound stain was solubilized with unbuffered tris base (100 μL, 10 mM). Cell density was determined using a fluorescence microplate reader (wavelength 540 nm). The obtained data were expressed as percentages of viability of treated cells versus negative control, for which viability was considered 100%. All measurements were replicated three times. Finally, from a plot of viability percentage against cannabis oil concentration, IC_50_ values were obtained by fitting the data to dose–response curves (Sigma Plot, Systat^®^ Software, Inc). The selectivity index (SI) of each extract was calculated as the ratio of IC_50_ (MCF-10A)/IC_50_ (cancer cell lines). If the values of SI were equal or greater than 2, it was assumed that the extract was selective.

## 4. Conclusions

Different varieties of *C. sativa* identified by the ratio of THC:CBD were used to extract cannabis oil using two extraction methods. The evaluation of the biological activities of the oils indicates that they are mostly determined by their chemical composition. For example, all *Cannabis* oils exhibit an antioxidant capacity and antiproliferative effects on tested cancer cell lines. In both types of experiments, the most active *Cannabis* oil tested was M4, suggesting a direct relationship between its antioxidant capacity and cancer cell cytotoxicity. In addition, M4 exhibits a high selectivity against breast cancer cell lines, and, therefore, *Cannabis* oils can be considered potential anticancer agents. This oil was obtained by SLE, and it contains high amounts of THC and CBD and the highest total content of phenolic and flavonoids compounds. Thus, its highest biological activities can be attributed to the entourage effect of CBD and its flavonoids compounds. Future work will focus on selecting different varieties of *C. sativa* but using a chemovar that gives more specific information about phenolic and flavonoids content in addition to the THC/CBD ratio.

The extraction of inflorescences using the SLE method with a polar solvent would provide a way to obtain *Cannabis* oils that could be used in the development of antifungal, antioxidant and anticancer agents.

## Figures and Tables

**Table 1:** Quantification of cannabinoids (Δ9-THC and CBD) using HPLC-UV in oils of inflorescences collected from different varieties of *C. sativa* L. Extraction methods: A: SC-CO_2_; B: SLE.

Variety *C. sativa* L.	Oil Sample	Extraction Method	Extraction Yield (wt %)	THC (mg/g Oil)	CBD (mg/g Oil)	Ratio THC/CBD
Critical+	M1	A	10.7	677 ± 50	<1.5	1:0.002
	M2	B	11.4	612 ± 24	32 ± 2.0	1:0.05
Shark Shock CBD	M3	A	12.2	255 ± 8.0	352 ± 7.0	0.72:1.0
	M4	B	13.1	254 ± 2.0	439 ± 3.0	0.58:1.0
Dinamed CBD	M5	A	9.6	22 ± 2.0	508 ± 23.0	0.04:1.0
	M6	B	11.2	6.5 ± 0.4	89 ± 4.0	0.07:1.0

**Table 2:** Total content of phenols, flavonoids and anthraquinones in essential oils extracted by SC-CO_2_ (A) and SLE (B) methods from female inflorescences that were collected from different varieties of *C. sativa* L. Concentrations are expressed as equivalents (mg/g of dried oil) of gallic acid (GAE), quercetin (QE) and emodin (EE).

Sample	Extraction Method	Total Phenols (GAE mg/g)	Total Flavonoids (QE mg/g)	Total Anthraquinones (EE mg/g)
M1	A	90.16 ± 6.80 ^a^	3.63 ± 0.09 ^a^	5.00 ± 0.05 ^a^
M2	B	102.07 ± 1.70 ^a^	6.32 ± 0.21 ^b^	5.38 ± 0.03 ^b^
M3	A	91.86 ± 1.70 ^a^	5.23 ± 0.06 ^c^	6.19 ± 0.03 ^c^
M4	B	91.86 ± 3.40 ^a^	8.19 ± 0.06 ^d^	6.05 ± 0.03 ^d^
M5	A	57.84 ± 3.40 ^b^	3.96 ± 0.24 ^e^	5.35 ± 0.03 ^e^
M6	B	78.26 ± 3.40 ^b^	9.10 ± 0.06 ^f^	6.32 ± 0.03 ^f^

Different letters in the same column indicate significant differences. *p* < 0.05; *n* = 3.

**Table 3:** Antioxidant capacity of essential oils extracted by SLE and SC-CO_2_ methods from female inflorescences collected from different varieties of *C. sativa* L. Last column lists EC_50_ values for antifungal activity against *T. mentagrophytes.*

Oil Sample	DPPH (IC_50_ mg/mL)	ABTS (TEAC mM)	FRAP (TEAC mM)	Antifungal Test EC_50_ (µg/mL)
M1	12.18 ± 0.19 ^a^	0.56 ± 0.09 ^a^	0.0120 ± 0.0003 ^a^	>240
M2	14.34 ± 0.38 ^b^	0.75 ± 0.02 ^b^	0.0209 ± 0.0007 ^a^	212.53 ± 2.27 ^c^
M3	14.68 ± 0.28 ^b^	0.71 ± 0.01 ^b^	0.0173 ± 0.0002 ^a^	>240
M4	12.80 ± 0.06 ^a^	0.81 ± 0.02 ^b^	0.0271 ± 0.0002 ^a^	89.37 ± 2.77 ^a^
M5	18.38 ± 0.42 ^c^	0.61 ± 0.03 ^c^	0.0131 ± 0.0007 ^b^	123.10 ± 3.03 ^b^
M6	13.28 ± 0.51 ^b^	0.72 ± 0.03 ^b^	0.0301 ± 0.0002 ^a^	161.56 ± 2.19 ^b^
Gallic Acid	n.a.	1.14 ± 0.01 ^d^	1.73 ± 0.026 ^c^	n.a.
BHT	0.06 ± 0.00 ^d^	1.06 ± 0.03 ^d^	1.53 ± 0.08 ^d^	n.a.
TROLOX^®^	0.11 ± 0.00 ^e^	1.0	n.a.	n.a.
Fluconazole^®^	n.a.	n.a	n.a	220 ± 1.23 ^c^

Different letters in the same column indicate significant differences. *p* < 0.05; *n* = 3; n.a: not applicable.

**Table 4:** Cytotoxic effect (IC_50_ μg/mL) and selectivity index (SI) of *C. sativa* oils against different cancer cell lines.

	IC_50_ (µg/mL)				SI		
Extracts	MCF-10	MCF-7	HT-29	PC-3	MCF-7	HT-29	PC-3
M1	61.2 ± 21.5	60.2 ± 16.9	62.6 ± 1.3	64.2 ± 0.7	1	1	1
M2	70.3 ± 4.1	18.0 ± 1.5	17.7 ± 1.4	21.0 ± 1.0	4	4	3
M3	30.9 ± 8.1	26.7 ± 1.0	37.2 ± 2.2	43.2 ± 0.9	1	1	1
M4	60.4 ± 25.3	13.0 ± 0.9	18.4 ± 1.0	21.9 ± 0.4	5	3	3
M5	61.8 ± 1.4	36.8 ± 1.5	47.8 ± 2.5	59.9 ± 15.2	2	1	1
M6	35.7 ± 6.2	15.4 ± 0.8	19.6 ± 0.9	22.5 ± 0.6	2	2	2

## Data Availability

Not applicable.

## Supplementary Materials

The following are available online at https://www.mdpi.com/article/10.3390/plants12091772/s1. Figure S1: Chromatograms of Cannabis sp. samples (M1–M6) analyzed by high-performance liquid chroma-tography coupled with ultraviolet spectroscopy (HPLC-UV). The quantification of THC and CBD at their respective retention times of 8 and 10 min, respectively. Figure S2: Plots of cell viability percentage of MCF-7, a breast cancer cell line, in the presence of different cannabis essential oils: (a) M2, (b) M4), (c) M6, and (d) MCF-10A (control) cell lines in the presence of M2.
