# Supplementary material for: Study of Cannabis Oils Obtained from Three Varieties of C. sativa and by Two Different Extraction Methods: Phytochemical Characterization and Biological Activities
Source: Plants (Basel). 2023 Apr 26;12(9):1772. doi: 10.3390/plants12091772 (PMC10180737; doi:10.3390/plants12091772)
Supplement: Supplementary file 1 [file plants-12-01772-s001.zip › plants-2374404-supplementary.pdf]

plants

Article

Study of Cannabis Oils Obtained from Three Varieties of *C. sativa* and by Two Different Extraction Methods: Phytochemical Characterization and Biological Activities

Sebastián Pino [1], Luis Espinoza [2], Carlos Jara-Gutiérrez [3], Joan Villena [3], Andrés F. Olea [4,*] and Katy Díaz [2,*]

[1] LABSUN (Laboratorio Sustentable Natural), Valparaíso 2340000, Chile; sebastian.pino@alumnos.usm.cl
[2] Departamento de Química, Universidad Técnica Federico Santa María, Avenida España 1680, Valparaíso 2340000, Chile; luis.espinozac@usm.cl
[3] Laboratorio de Investigación-Estrés Oxidativo, Centro de Investigaciones Biomédicas (CIB), Facultad de Medicina, Universidad de Valparaíso, Viña del Mar 2520000, Chile; carlos.jara@uv.cl (C.J.-G.); juan.villena@uv.cl (J.V.)
[4] Grupo QBAB, Instituto de Ciencias Químicas Aplicadas, Facultad de Ingeniería, Universidad Autónoma de Chile, El Llano Subercaseaux 2801, Santiago 8900000, Chile
* Correspondence: andres.olea@uautonoma.cl (A.F.O.); katy.diaz@usm.cl (K.D.); Tel.: +56-32-2652843 (K.D.)

Supplementary Materials: **The following are available online at www.mdpi.com/xxx/s1, Figure S1**: Chromatograms of *Cannabis* sp. samples (**M1-M6**) analyzed by high-performance liquid chromatography coupled with ultraviolet spectroscopy (HPLC-UV), quantification of THC and CBD at their respective retention times 8 and 10 min respectively; **Figure S2:** Viability percentage of MCF-7, a breast cancer cell line in presence of different cannabis essential oils a) M2, b) M4), c) M6, d) MCF-10A (control) cell lines in presence of M2.

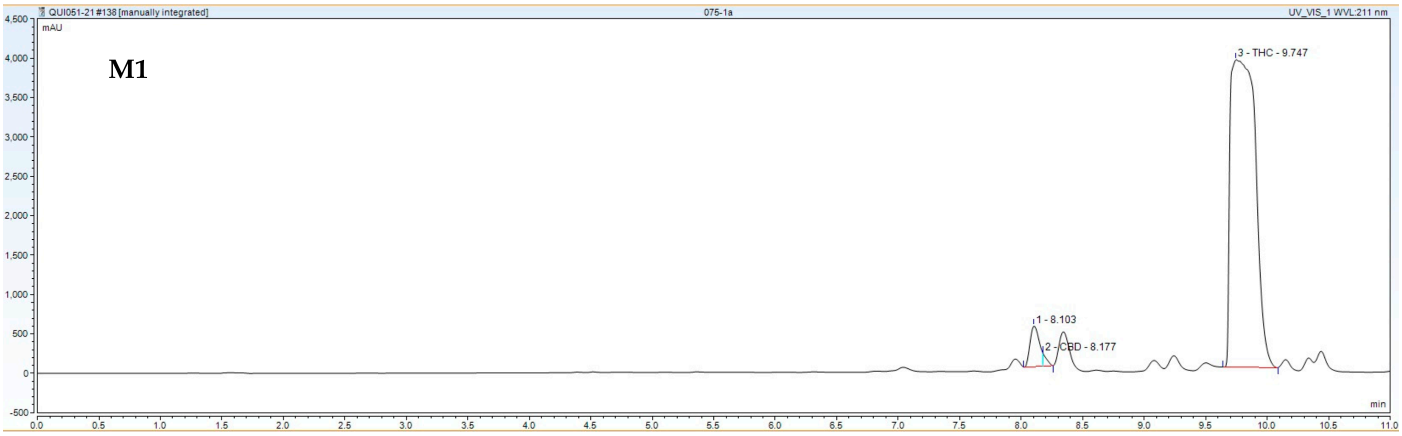
QUI051-21 #138 [manually integrated]
075-1a
UV_VIS_1 WVL:211 nm
mAU
M1
3 - THC - 9.747
1 - 8.103
2 - CBD - 8.177
min

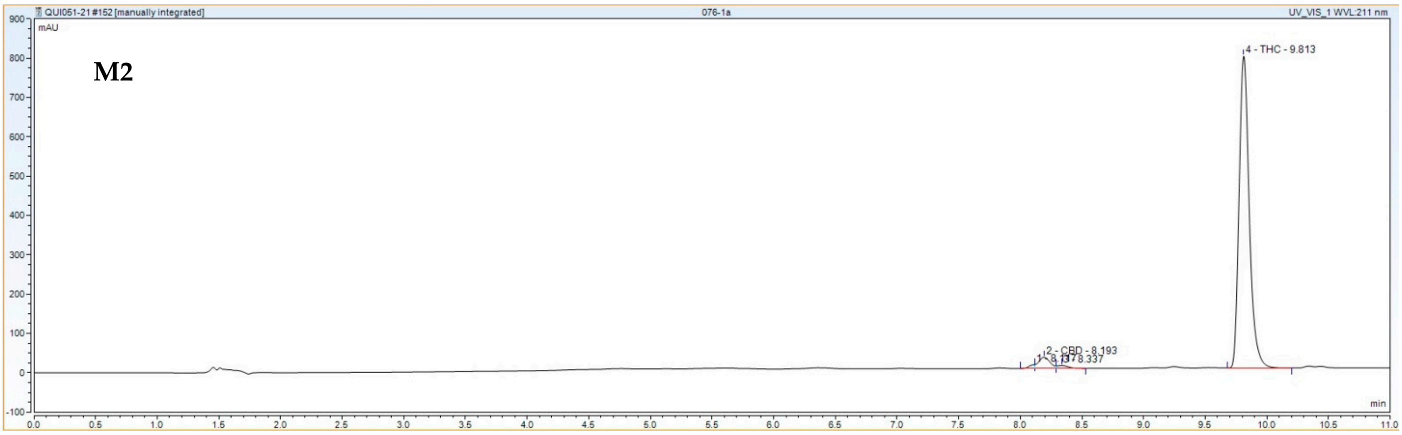
QUI051-21 #152 [manually integrated]
076-1a
UV_VIS_1 WVL:211 nm
mAU
M2
4 - THC - 9.813
2 - CBD - 8.193
min

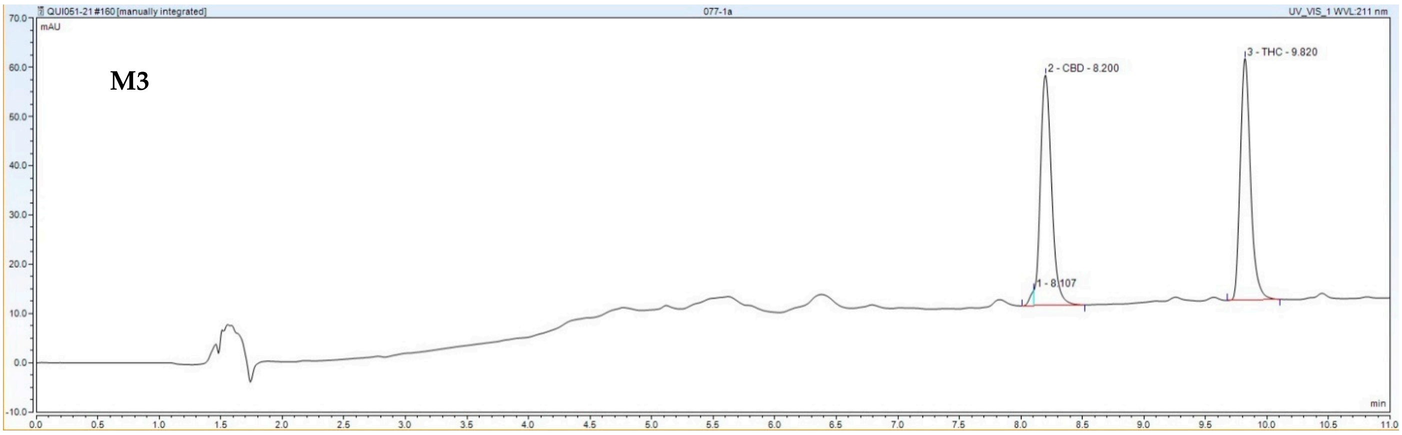
QUI051-21 #160 [manually integrated]
077-1a
UV_VIS_1 WVL:211 nm
mAU
M3
2 - CBD - 8.200
3 - THC - 9.820
1 - 8.107
min

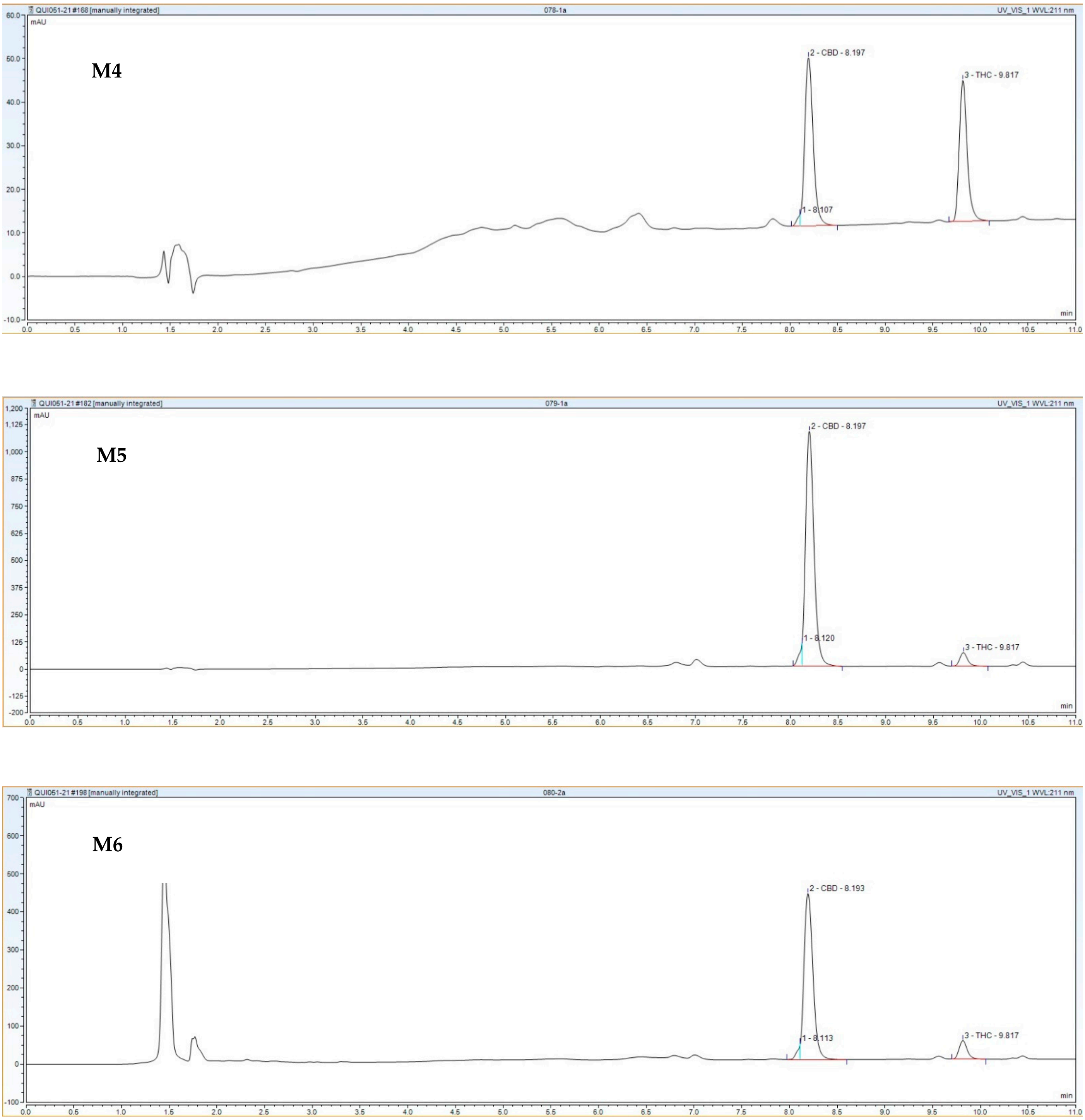

Figure S1: Chromatograms of Cannabis sp. samples (**M1-M6**) analyzed by high-performance liquid chromatography coupled with ultraviolet spectroscopy (HPLC-UV), quantification of THC and CBD at their respective retention times 8 and 10 min respectively.

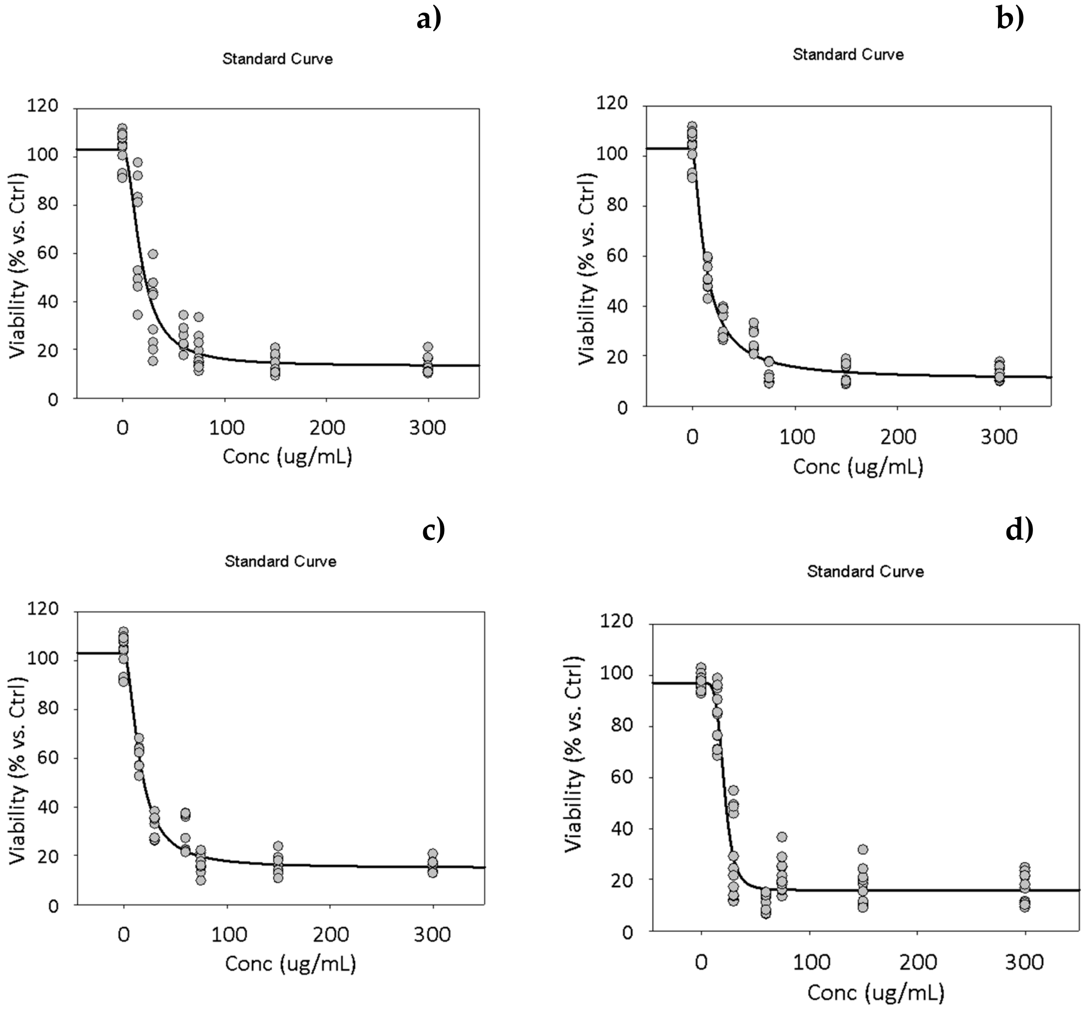

Figure S2. Viability percentage of MCF-7, a breast cancer cell line in presence of different cannabis essential oils **a)** M2, **b)** M4), **c)** M6, **d)** MCF-10A (control) cell lines in presence of M2.
